# Characterization of Stimulated γδ T Cells: Phenotypic Analysis and Implications for Allogeneic Cellular Immunotherapy

**DOI:** 10.3390/cells14231917

**Published:** 2025-12-02

**Authors:** Anna Bold, Heike Gross, Marco Bardenbacher, Elisabeth Holzmann, Stefan Knop, Martin Wilhelm

**Affiliations:** Department of Hematology and Medical Oncology, Paracelsus Medical University, 90419 Nuremberg, Germany

**Keywords:** γδ T cells, Vy9Vδ2 T cells, cellular immunotherapy, zoledronate, interleukin 2, surface molecules, activation, phenotype

## Abstract

**Highlights:**

**What are the main findings?**

- Provision of a phenotypic analysis of γδ T cells at the time of isolation and after ten days of stimulation using a protocol based on zoledronate and interleukin 2.
- Identification of potential phenotypic markers predictive of donor suitability for allogeneic cell therapies.

**What are the implications of the main findings?**

- Contribution to the understanding of γδ T cell biology and their therapeutic potential.
- Basis for future clinical trials.

**Abstract:**

Due to their anti-tumor activity and non-major histocompatibility complex (MHC) binding T cell receptor, γδ T cells are suitable candidates for allogeneic cellular immunotherapy in cancer. Recently, we developed a new protocol called Ko-Op for stimulation of γδ T cells (specifically Vy9Vδ2 T cells) that generates a cell product consisting mainly of γδ T cells with preserved anti-tumor activity targeted for clinical-grade application. In this study, we investigated the phenotype of stimulated γδ T cells and correlated this with results of functional assays to obtain a deeper understanding of the characteristics of stimulated γδ T cells. Additionally, an intensive analysis of surface molecules of unstimulated and stimulated γδ T cells is presented. Since heterogeneous results regarding the response to therapy with γδ T cells observed in earlier clinical trials could be a consequence of various extents of γδ T cell adhesion and migration ability, we addressed surface molecules associated with cellular activity and adhesion and migration functions as well. By investigating correlations between the phenotype of unstimulated γδ T cells and cellular cytotoxicity, as well as the degranulation ability of stimulated γδ T cells, we could draw conclusions about optimal donors for further allogeneic cellular therapies. Finally, we demonstrated that the phenotype varies over the time of culture and is clearly modifiable by changing the stimulation protocol.

## 1. Introduction

γδ T cells are promising candidates for cellular immunotherapy of cancer. Their anti-tumor effects have been demonstrated in a comprehensive way in vitro and in vivo [1,2,3,4]. The key advantage of these cells is the possibility of allogeneic transfer without graft versus host reaction as shown in several clinical studies [5,6,7,8]. For immunotherapy of cancer, γδ T cells can be stimulated by aminobisphosphonates or natural or synthetic phosphoantigens, known to stimulate the Vy9Vδ2 T cells, and an addition of co-stimulators like interleukin 2 (IL-2) [9,10,11]. Recently, we developed a new protocol for ex vivo stimulation that generates a cell-rich cell product mainly consisting of γδ T cells with preserved anti-tumor activity within ten days and that allows for easy transition to future Good Manufacturing Practice (GMP) compliant processing [12].

The aim of this study is to characterize the stimulated γδ T cells more precisely on the basis of their morphology and the expression levels of surface molecules and to compare it with unstimulated γδ T cells. Despite promising in vitro results, the response of cancer patients to the cellular therapy with γδ T cells in clinical studies has been very heterogeneous [13]. A possible cause of inadequate response to immunotherapy with γδ T cells may be the inefficient tumor infiltration and persistence of γδ T cells [14]. Therefore, we determined not only molecules that are associated with stimulation and anti-tumor activity but also various adhesion and migration molecules on the assumption that increased expression may be associated with improved tumor infiltration. Among others, we determined CD44, CD162 (P-Selectin Glycoprotein Ligand-1), and the chemokine receptors CXCR3 and CXCR4. As previously reported, CXCR3 is highly expressed on Vy9Vδ2 T cells of healthy donors [15]. As its expression is also associated with a Th1 like phenotype and CXCR3 is shown to promote migration of T cells into tumor tissue in vitro, it is a relevant surface molecule for evaluating the results of our stimulation procedure [16,17]. CXCR4 is known to be involved in transendothelial migration of γδ T cells [18]. In contrast, very little is known about CD162 expression on γδ T cells in the peripheral blood. However, the migration of activated T cells to inflammatory tissue is shown to be regulated by CD162 and CD44 by binding E-Selectin [19]. We also tested surface molecules associated with (anti-tumor) activity. CD16 (FcyRIIIA) is a type I transmembrane receptor which is known to mediate antibody-dependent cellular cytotoxicity (ADCC) by natural killer (NK) and T cells. CD56, also named the neural cell adhesion molecule, is expressed on the cell surface of a number of immune cells such as NK cells, T cells, or dendritic cells [20]. It has been shown several times that CD56 expression on γδ T cells increases by stimulation and that the expression of CD56 is associated with anti-tumor cytotoxicity [21,22]. CD154, also called CD40 ligand, is a membrane protein that binds to CD40 on antigen-presenting cells and is known to be upregulated on activated CD4^+^ T cells and γδ T cells [23]. Furthermore, we examined unstimulated and stimulated γδ T cells for early activation markers such as CD25 and CD69. Among other functions, CD69 is required for the recruitment of resident T cells in epithelial and mucosal tissues [24]. Although Vy9Vδ2 T cells are present primarily in peripheral blood and not in epithelia, recent data show that CD69^+^ Vδ2 T cells migrate into tissues and exert anti-inflammatory effects [25].

To classify γδ T cells in terms of their function, they can be divided into naive, central memory (TCM), effector memory (TEM), and terminally differentiated (TEMRA) γδ T cells based on the expression of CD45RA and CD27 [26]. A memory phenotype seems to be beneficial for persistence of CAR T cells and therefore for response to that therapy [27]. Additionally, we know from CD8^+^ T cell studies, that a central memory subset expressing the chemokine receptor CCR7 (CD197) and CD62L (L-Selectin) has been shown to be more effective at controlling tumor in vivo than an effector memory subset [28,29,30]. Thus we also investigated the subtypes and the CCR7 and CD62L expression here.

Immune checkpoints, whose upregulation on T cells can be considered an indication of exhaustion, are also examined here [31]. In addition to well-known immune checkpoints, such as PD-1 (programmed death 1), TIGIT (T cell immunoreceptor with Ig and ITIM domains), LAG-3 (lymphocyte-activation gene 3), and TIM-3 (T cell immunoglobulin and mucin-domain containing 3) are determined as well.

Other surface molecules not listed here were investigated as part of this study. Although the expression of some surface molecules on γδ T cells has been determined in previous studies [32,33], an intensive analysis comparing unstimulated and stimulated γδ T cells especially including surface molecules with adhesion and migration function, has not been performed to our knowledge.

The purity of the stimulated γδ T cells obtained by stimulation with the Ko-Op protocol is high regardless of different donors, but the proliferation rate and anti-tumor activity varies from donor to donor, observed with other stimulation methods as well [34,35]. In our previous study, we have already seen evidence that the cells from younger donors with a higher rate of γδ T cells in the peripheral blood can be stimulated more effectively [12]. Here, we correlated the expression of surface molecules of unstimulated γδ T cells with the results of functional assays with the stimulated γδ T cells from the same donors to evaluate if the phenotype can serve as a criterion for selection of donors.

We further aimed to investigate whether the cultivation procedure influences the γδ T cells phenotype. Thus, we compared the expression of the surface molecules of γδ T cells stimulated with Ko-Op with the expression of surface molecules on γδ T cells stimulated with a non-GMP-compliant, standard protocol for stimulating γδ T cells. This protocol leads to a less pure cell product with reduced anti-tumor activity compared to the simulation with Ko-Op [12].

## 2. Materials and Methods

### 2.1. Cell Culture, Cell Isolation, and Ex Vivo Stimulation of γδ T Cells

Our investigations were performed with 20–40 mL peripheral blood obtained from healthy adult donors. The studies involving human participants were reviewed and approved by the Institutional Review Board of the Paracelsus Medical University Nuremberg and by the ethics committee of the Bavarian State Medical Association (EC number: 23053). Written informed consent to participate in this study was provided by the participants. All donors signed an agreement according to General Data Protection Regulation.

Peripheral blood mononuclear cells (MNC) were isolated by density gradient centrifugation with Biocoll (Biochrom, Darmstadt, Germany/Bio&SELL, Feucht, Germany) and if indicated divided into two fractions in order to expand them using different cultivation methods. MNC were incubated for up to 17 days at 37 °C and 5% CO_2_ in both protocols.

For culture according to the Ko-Op protocol [12], MNC at a concentration of 5 × 10^5^/mL were cultured in 50 mL cell culture flasks (Sarstedt, Nuembrecht, Germany) using a medium consisting of OpTmizerTM CTSTM T Cell Expansion Basal Medium, OpTmizerTM CTSTM T Cell Expansion Supplement (Gibco/Thermo Fisher, Waltham, MA, USA), and 1% 200 mM L-glutamine (Biochrom, Darmstadt, Germany/Bio&SELL, Feucht, Germany). On day 0, 10 µM zoledronate (Sigma-Aldrich, St. Louis, MO, USA) was added and 1000 U/mL interleukin 2 (IL-2) (Burton-on-Trent, Great Britain, United Kingdom) was added on day 2. On day 4, 7, 9, 11, and 14 half of the medium was removed and replaced by fresh medium containing 2 × 1000 U/mL IL-2 for a final concentration of 1000 U/mL. Cells were not split at any time. From day 4 onwards, cell culture flasks were shaken at 250 rpm.

For culture according to the R10F protocol, MNC at a concentration of 5 × 10^5^/mL were cultured in 96 U-bottom plates (Greiner bio-one, Frickenhausen, Germany) using standard medium consisting of RPMI 1640 supplemented with 10% fetal bovine serum (FCS), 1% 200 mM L-glutamine, and 1% penicilline/streptomycin (all from Biochrom, Darmstadt, Germany/Bio&SELL, Feucht, Germany). On day 0, 1 µM zoledronate and 100 U/mL IL-2 were added. From day 7, the cells were harvested twice a week, washed, and reseeded at a concentration of 5 × 10^5^/mL in 96 well plates with fresh medium and 100 U/mL IL-2.

The lymphoma cell lines Daudi, Mino, and U2932 were obtained from the German collection of microorganisms and cell cultures (DSMZ, Braunschweig, Germany) and cultured in our standard medium R10F.

Cell counts and cell viability were established using a hemocytometer and the trypan blue exclusion method. Cell proliferation rate of MNC was calculated based on the cell concentrations at day 0 (always set to 5 × 10^5^/mL) and day 10 of stimulation by the following formula: Proliferation rate = cell concentration (d10)/cell concentration (d0).

### 2.2. Flow Cytometry and Antibodies

A FC500 and a Navios flow cytometer (both Beckman Coulter, Brea, CA, USA) were used for multicolor immunofluorescence and functional tests. For measurement of surface molecules, 2 × 10^5^ MNC were stained and 3 × 10^3^ γδ T cells in unstimulated MNC and 1 × 10^4^ γδ T cells in stimulated MNC were acquired.

Cells were stained in appropriate combinations according to use with following antibodies: anti-T cell receptor (TCR) γδ-fluorescein isothiocyanate (FITC) [clone IMMU510], anti-CD3-r-Phycoerythrin-Texas Red (ECD) [clone UCHT1], anti-CD56-phycoerythrin-cyanine 5 (PC5) [clone N901], anti-CD16-PC5 [clone 3G8], anti-CD27-PC5 [clone 1A4CD27], anti-CD56-phycoerythrin-cyanine 7 (PC7) [clone N901] (all Beckman Coulter, Brea, CA, USA); anti-T cell receptor (TCR) γδ-FITC [clone 11F2], anti-CD107a-phycoerythrin (PE) [clone H4A3], anti-IFNγ-PE [clone 45-15], anti-CD57-PE [clone REA 769], anti-CCR7-PE [clone FR 11-11E8], anti-CD45RA-PE [clone REA 562], anti-TIGIT-allophycocyanin (APC) [clone REA 1004], anti-CD337(NKp30)-APC [clone REA823], anti-CD336(NKp44)-APC [clone REA1163], anti-CD226(DNAM-1)-APC [clone REA1040], anti-CD184(CXCR4)-APC [clone 12G5] (all Miltenyi Biotec, Bergisch Gladbach, Germany); anti-CD25-PE [clone BC96], anti-CD28-PE [clone CD28.2], anti-CD44-PE [clone BJ18], anti-CD69-PE [clone FN50], anti-CD162-PE [clone KPL-1], anti-CD183(CXCR3)-PE [clone G025H7], anti-CD279(PD-1)-PE [clone EH12.2H7], anti-335(NKp46)-PE [clone 9E2], anti-CD62L-PC5 [clone DREG-56], anti-CD69-PC5 [clone FN50], anti-CD154-PC5 [clone 24-31], anti-CD11aCD18(LFA-1)-PC7 [clone m24], anti-CD103- PC7 [clone Ber-ACTG8], anti-CD152(CTLA-4)- PC7 [clone L3D10], anti-CD192(CCR2)-PC7 [clone K036C2], anti-CD223(LAG-3)-PC7 [clone 11C3C65], anti-CD314(NKG2D)-PC7 [clone 1D11], anti-CD31-pacific blue (PB) [cloneWM59], anti-CD161-PB [clone HP-3G10], and anti-CD366(TIM-3)-PB [clone F38-2E2] (all Biolegend, San Diego, CA, USA). The antibody used to detect LFA-1 (clone m24) binds the high-affinity conformation of CD11a/CD18 and thus specifically recognizes the active state of LFA-1 [36].

To define the percentage of γδ T cells expressing the respective surface molecule, cells were stained with an isotype control and the gate was adjusted so that 2% of the cells in the isotype control were defined as positive. ΔMFI (median fluorescence intensity) was calculated as MFI (CD107a) minus MFI (isotype control). As isotype control, anti-IgG1-PE [clone IS5-32F5], anti-REA(S)-PE [clone REA293], and anti-REA(S)-APC [clone REA293] (all Miltenyi Biotec, Bergisch Gladbach, Germany), as well as anti-IgG1-PC5 [clone MOPC-21], anti-IgG1-PC7 [clone MOPC-21], anti-IgG2a-PC7 [clone MOPC-173], anti-IgG2a-APC [clone MOPC-173], and anti-IgG1-PB [clone MOPC-21] (all Biolegend, San Diego, CA, USA) were used.

γδ T cells were defined as CD3^+^ TCR γδ^+^ MNC, NK cells as CD3^−^ CD56^+^ MNC, and αβ T cells as CD3^+^ TCR γδ^−^ MNC. An anti-pan γδ TCR was used for this study. Therefore, we refer to γδ T cells in general in the manuscript, although the Ko-Op protocol primarily stimulates Vδ2 T cells.

Subgroups of γδ cells based on their CD45RA and CD27 expression were determined according to Dieli et al. as the following: naive γδ T cells were defined as CD45RA^+^ CD27^+^ γδ T cells, central memory γδ T cells (TCM) as CD45RA^−^ CD27^+^ γδ T cells, effector memory γδ T cells (TEM) as CD45RA^−^ CD27^−^ γδ T cells, and terminally differentiated γδ T cells (TEMRA) as CD45RA^+^ CD27^−^ γδ T cells [26].

For the cytotoxicity assay, the therapy grade monoclonal antibody rituximab (Roche, Grenzach-Wyhlen, Germany) as well as the IgG1, kappa control antibody (Sigma-Aldrich, St. Louis, MO, USA) were used.

### 2.3. Microscopy of Unstimulated and Stimulated γδ Cells

To facilitate adherence of MNC, 12 mm microscope coverslips were pre-coated with poly-D-lysine. For this, coverslips were sterilized by incubation in 70% (*v*/*v*) isopropanol. After drying, coverslips were placed in 24-well flat-bottom plates (Greiner Bio-One, Frickenhausen, Germany) and incubated with 0.1 mg/mL poly-D-lysine (Gibco, Thermo Fisher Scientific, Waltham, MA, USA) for 1 h. Afterwards, coverslips were washed three times with deionized water, allowed to dry, and stored at 4 °C until use.

Either freshly isolated MNC at day 0 or MNC stimulated according to the Ko-Op protocol for 10 days were seeded on coverslips and allowed to adhere for 2 h. Cells were fixed using 4% paraformaldehyde (Otto Fischar, Saarbrücken, Germany), and blocking was performed with 10% fetal calf serum (Bio&SELL, Feucht, Germany) in PBS at room temperature. Antibodies (anti-TCRγδ-allophycocyanin [clone 11F2] (Miltenyi Biotec, Bergisch Gladbach, Germany) and phalloidin-iFluor 488 reagent for staining of actin (Abcam, Cambridge, UK)) were applied overnight at 4 °C. Counterstaining with DAPI (Sigma-Aldrich, St. Louis, MO, USA) was performed before mounting. Slides were visualized using a Zeiss Axiovert 5 widefield microscope (Carl Zeiss, Oberkochen, Germany).

Quantitative analyses of cell area and perimeter were obtained using a custom CellProfiler 4.2.8 pipeline for automated quantification [37]. Nuclei were first segmented using DAPI staining, followed by the identification of γδ T cells via TCRγδ staining. Cell area and perimeter of the identified γδ T cells were quantified based on actin staining.

### 2.4. Degranulation Assay

The surface antigen CD107a as marker for degranulation was determined in our degranulation assay. For this, stimulated MNC (Ko-Op protocol) were co-cultured with Daudi, Mino, or U2932 in a 1:2 ratio or with the respective medium with the addition of the anti-CD107a-PE or anti-IgG-PE as control in 96 well V-bottom plates (Greiner bio-one, Frickenhausen, Germany) for 3 h. Subsequently, the cells were washed, the surface antigens were stained with anti-TCR γδ-FITC and anti-CD3-ECD and the cells were analyzed by flow cytometry. To define CD107a^+^ γδ T cells, cells were stained with an isotype control and the gate was adjusted so that 2% of the cells in the isotype control were defined as positive. ΔMFI was calculated as MFI (CD107a) minus MFI (isotype control).

### 2.5. Cytotoxicity Assay

Cytotoxicity experiments were conducted in 96-well V-bottom plates (Greiner bio-one, Frickenhausen, Germany) as co-cultures at different effector-to-target cell ratios (0.7:1, 2.2:1, 6.7:1, and 20:1) or as targets only with 1 µg/mL rituximab or IgG1, kappa as control. Daudi were used as target cells and previously labeled with carboxyfluorescein succinimidyl ester (CFSE) (BioLegend, San Diego, CA, USA) to be able to distinguish them from the effector cells after co-culture and taken up into the culture medium (OpTmizerTM CTSTM T Cell Expansion Medium). MNC were stimulated according to the Ko-Op protocol for ten days and used as effector cells in this assay. Co-cultures were plated as triplicates. Following co-culture of effector and target cells for 4 h, cells were harvested, technical replicates were pooled, treated with 7-AAD (Beckman Coulter, Brea, CA, USA) and analyzed by flow cytometry. Specific cell-mediated cytotoxicity is expressed as “specific lysis %” and calculated by the formula: specific lysis % = [% 7-AAD^+^ target cells in the respective effector-to-target ratio − % 7-AAD^+^ target cells in target cell only culture] × 100/[100 − % 7-AAD^+^ target cells in target cell only culture]. The lytic units per 10^6^ effector cells were calculated according to Bryant et al. with the following formula: $lytic\;units\;per\;10^{6}\;effector\;cells=10^{6}\cdot exp\left(\frac{\bar{Y^{*}}-\;Y_{p}^{*}}{C}\right)/(T\cdot \bar{X_{G}}$) [38]. *Y* is the specific lysis measured in a defined effector-to-target ratio. *Y* is logistically transformed ($Y^{*}$) by the following formula: $Y^{*}=LN(\frac{Y}{100-Y})$. We defined a reference lysis p of 60%, which is also logistically transformed ($Y_{p}^{*}).$ $\bar{Y^{*}}$ is the arithmetic mean of the logistically transformed specific lyses measured in each effector-to-target ratio, C is a constant with relation to the slope of the curve (defined as 1), T is the number of target cells $\left(10^{4}\right)$, and $\bar{X_{G}}$ is the geometric mean of the effector-to-target ratios used in the assay.

### 2.6. Data and Statistical Analysis

Data were analyzed with the software Kaluza analysis V2.1 (Beckman Coulter, Brea, CA, USA), Excel 2016 (Microsoft, Redmond, WA, USA), SPSS Statistics 22 (IBM, Armonk, NY, USA), and GraphPad Prism Version 10.4.1 (Graphpad Software, Boston, MA, USA). Data are presented as mean ± standard deviation (SD). The normal distribution of the data was verified using the Shapiro test. Levels of significance were calculated using the paired or unpaired *t*-test or the Wilcoxon test. Correlation coefficient R was calculated according to Spearman. *p* < 0.05 is considered statistically significant.

## 3. Results

### 3.1. Phenotype of Unstimulated and Stimulated γδ T Cells

**Figure 1:**
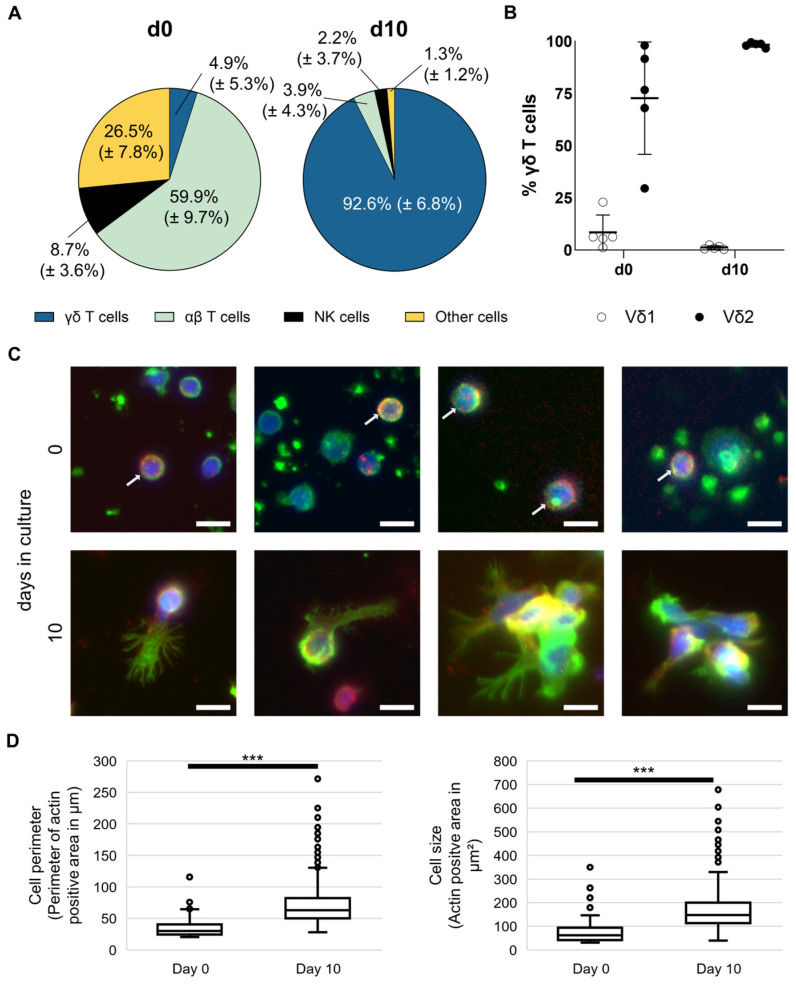
Composition and morphology of unstimulated and stimulated γδ T cells. MNC of healthy donors were isolated and stimulated according to the protocol Ko-Op. (**A**) Composition of MNC at day 0 and day 10 of culture determined by flow cytometry. (**B**) Percentage of Vδ1 and Vδ2 T cells of all γδ T cells at day 0 and day 10 of culture determined by flow cytometry. (**C**) Representative widefield fluorescence images of cells stained for F-actin (green), TCRγδ (red), or DAPI (blue) at day 0 or day 10 of culture. White arrows mark cells positive for TCRγδ at day 0. Bars equal 10 µm. (**D**) Perimeter and area of TCRγδ^+^ cells were quantified based on F-actin immunofluorescence measured at day 0 and day 10 of culture. Per donor, 21.5 ± 10.3 cells at day 0 and 400.5 ± 159.4 cells at day 10 were evaluated, resulting in total sample sizes of 86 cells and 1602 cells, respectively. The data are presented as mean ± SD of 20 (**A**) or 5 (**B**) independent experiments or as a boxplot of 4 (**D**) independent experiments. *** *p* < 0.001 comparing unstimulated and stimulated γδ T cells.

MNC from 20 healthy donors have been isolated and stimulated according to the Ko-Op protocol for ten days. Percentage of γδ T cells, αβ T cells, and NK cells have been analyzed by flow cytometry on day 0 and day 10 and reveal a remarkable increase in the γδ T cell proportion (Figure 1A). Exemplary gating strategy for unstimulated and stimulated MNC is shown in Figure S1. Additionally, we analyzed the Vδ1 and Vδ2 subtypes of unstimulated and stimulated γδ T cells of five healthy donors. As expected, stimulation according to Ko-Op leads to stimulation of the Vδ2 subtype (Figure 1B). Next, we obtained microscopy images of unstimulated and stimulated γδ T cells from four different healthy donors cultured for 10 days according to the Ko-Op protocol. Therefore, cells were stained for γδ T cell receptor, DAPI, and F-actin in order to analyze cell form and structure. As exemplified in Figure 1C, the morphology of the γδ T cells changes dramatically, in that the size increases and cells change from a round to an elongated shape and form protrusions. Additionally, they tend to form aggregates as shown in the right pictures in the lower row (Figure 1C). The increase in size is statistically significant (Figure 1D).

**Figure 2:**
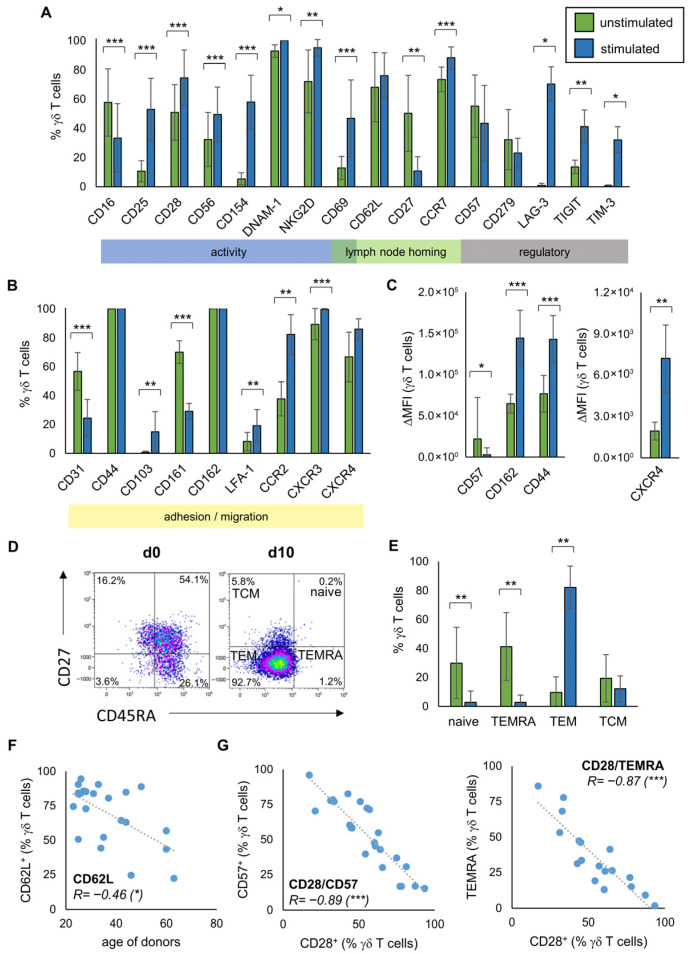
Phenotype of unstimulated and stimulated γδ T cells. MNC of healthy donors were isolated and stimulated according to the protocol Ko-Op. The surface molecules on unstimulated γδ T cells and after 10 days of stimulation were determined by flow cytometry. (**A**,**B**) Percentage of the respective surface molecule expressing γδ T cells unstimulated (green bars) and after ten days in culture (blue bars). The colored boxes show the main function of the molecules. (**C**) Expression of CD57, CD162, CD44, and CXCR4 (shown as ΔMFI) on γδ T cells unstimulated (green bars) and after ten days in culture (blue bars). (**D**) Exemplary gating strategy for determination of γδ T cells differentiated in naive (CD45RA^+^CD27^+^), TCM (CD45RA^−^CD27^+^), TEM (CD45RA^−^CD27^−^), and TEMRA (CD45RA^+^CD27^−^) at day 0 and day 10. (**E**) Percentage of γδ T cells differentiated in naive, TCM, TEM, and TEMRA unstimulated (green bars) and after ten days in culture (blue bars). (**F**) Correlation between the percentage of CD62L expressing unstimulated γδ T cells and the age of donors. (**G**) Correlation between the percentage of CD28 and CD57 expressing unstimulated γδ T cells and of CD28 expressing unstimulated γδ T cells and TEMRA. The data are presented as mean ± SD of 6–19 (**A**,**B**), 7–15 (**C**) or 14 (**E**) independent experiments, or as a correlation chart of 23 (**F**) or 18–24 (**G**) independent experiments. Correlation coefficient R is calculated according to Spearman. * *p* < 0.05, ** *p* < 0.01, and *** *p* < 0.001 comparing unstimulated and stimulated γδ T cells (**A**,**B**,**D**) or correlating the indicated variables (**F**,**G**).

Furthermore, we investigated the phenotype of γδ T cells with the expression of the different surface molecules serving as activity and adhesion markers measured by flow cytometry. For this, MNC from 19 healthy donors were isolated and stimulated according to the Ko-Op protocol. Figure 2A,B gives an overview of the expression of different surface molecules on unstimulated γδ T cells and after 10 days of stimulation, as measured by flow cytometry. The main function of these molecules is shown in the colored strips below. Exemplary flow histograms for every tested surface molecule are shown in Figures S2 and S3. While the percentage of γδ T cells expressing CD16, CD27, CD31, and CD161 decreases, the percentage of γδ T cells expressing CD25, CD28, CD56, CD154, DNAM-1, NKG2D, CD69, CCR7, LAG-3, TIGIT, TIM-3, CD103, CCR2, and CXCR3 increases significantly. Also the percentage of active LFA-1 is increased. The immune checkpoint surface molecule CD152 (CTLA-4) as well as the NK cell receptors NKp30, NKp44, and NKp46 are weakly expressed in both unstimulated and stimulated cells (Figure S3). There is a significant increase for CD44, CD162, and CXCR4 and a significant decrease for CD57 only when looking at the ΔMFI (Figure 2C). The distribution of the subtypes of naive γδ T cells, TCM, TEM, and TEMRA based on their CD45RA and CD27 expression is shown in Figure 2D as an exemplary flow plot. At day 10 of stimulation, the percentage of naive γδ T cells and TEMRA is significantly decreased compared to day 0, while the percentage of TEM increased significantly (Figure 2E). By investigating MNC from 23 healthy donors, we observed that donor age inversely correlated with the proportion of CD62L expressing γδ T cells in peripheral blood (Figure 2F). We also observed correlations regarding the expression of the individual surface molecules on peripheral blood γδ T cells from at least 18 healthy donors. Thus, the percentage of CD28 expressing γδ T cells negatively correlates with the percentage of CD57 expressing γδ T cells as well as with the percentage of TEMRA (Figure 2G). The percentage of CD57 expressing γδ T cells, in turn, is positively correlated with the percentage of TEMRA (Table S1A). Other significant correlations regarding the expression of surface molecules are shown in Table S1.

### 3.2. Relationship Between Phenotype of Stimulated γδ T Cells and Their Anti-Tumor Activity

**Figure 3:**
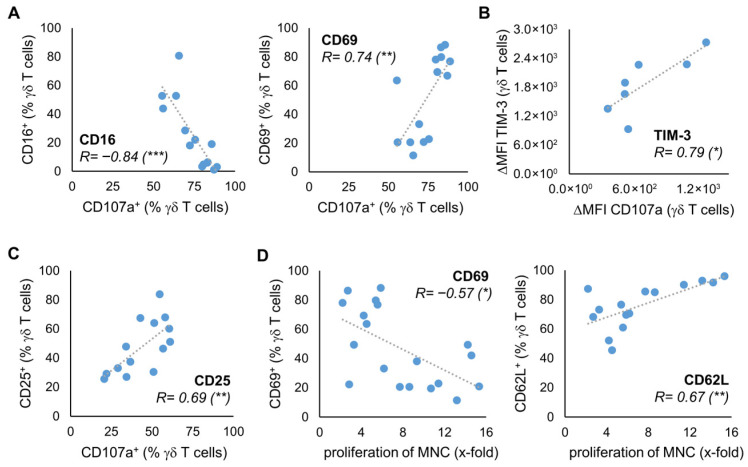
Relationship between phenotype and anti-tumor activity. MNC of healthy donors were isolated and stimulated according to the protocol Ko-Op. On day 10, the proliferation rate of the MNC was determined, a degranulation assay was performed, and the expression of surface molecules on γδ T cells was determined by flow cytometry. (**A**) Correlation between the percentage of CD16 or CD69 expressing γδ T cells and the percentage of CD107a expressing γδ T cells after incubation with Daudi for 3 h. (**B**) Correlation between the ΔMFI of TIM-3 on γδ T cells and the ΔMFI of CD107a on γδ T cells after incubation with U2932 for 3 h. (**C**) Correlation between the percentage of CD25 expressing γδ T cells and the percentage of CD107a expressing γδ T cells after incubation with medium control for 3 h. (**D**) Correlation between the percentage of CD69 or CD62L expressing γδ T cells and the proliferation rate of MNC. The data are presented as a correlation chart of 14 (**A**,**C**), 13 (**B**), or 15–19 (**D**) independent experiments. Correlation coefficient R is calculated according to Spearman. * *p* < 0.05, ** *p* < 0.01, and *** *p* < 0.001 correlating the indicated variables.

To investigate whether the expression of the surface molecules on stimulated γδ T cells is associated with the anti-tumor efficacy of the γδ T cells, we stimulated γδ T cells from 14 healthy donors according to the Ko-Op protocol. After ten days, we determined the proliferation rate and performed a degranulation assay with or without the lymphoma cell lines Daudi, Mino, and U2932 (Figure S4). Subsequently, we tested the results for correlation with the expression of the surface molecules at day 10. For CD16, we found a negative correlation between the percentage of CD16 expressing γδ T cells and the percentage of CD107a expressing γδ T cells after 3h incubation with Daudi. For CD69, there is a positive correlation (Figure 3A). There is also a positive correlation between the ΔMFI of TIM-3 on γδ T cells at day 10 and the ΔMFI of CD107a after incubation with Daudi and the percentage of CD25^+^ γδ T cells and CD107a^+^ γδ T cells after incubation with media control (Figure 3B,C). The significant correlations shown were observed with at least one other cell line as a target. Figure S5A provides an overview of the correlations found. Next, we investigated if the expression of certain surface molecules of stimulated γδ T cells from at least 15 healthy donors correlates with the proliferation rate of these cells. Interestingly, higher proliferation rate is negatively correlated with CD69 expression at day 10 (Figure 3D). In contrast, there is a positive correlation between the percentage of γδ T cells expressing CD62L and the proliferation rate at d10.

### 3.3. The Characteristics of Unstimulated γδ T Cells Correlate with Anti-Tumor Activity After Stimulation

**Figure 4:**
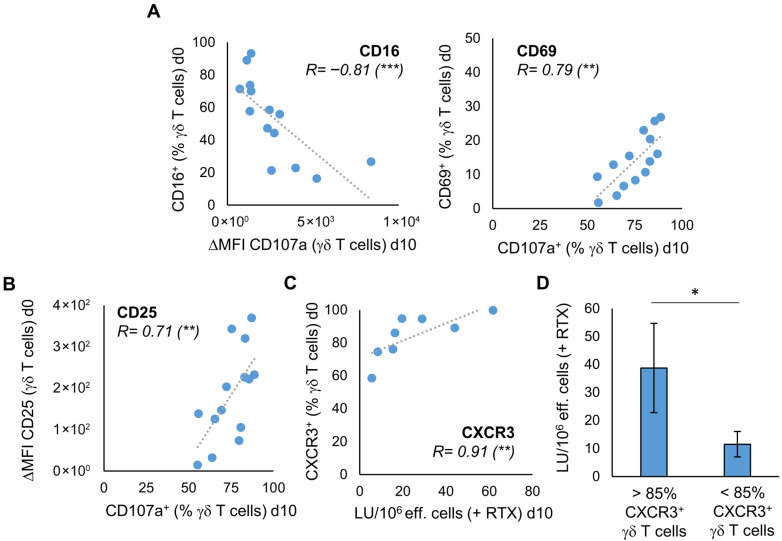
The characteristics of unstimulated γδ T cells correlate with their proliferation rate and anti-tumor activity after stimulation. MNC of healthy donors were isolated and stimulated according to the protocol Ko-Op. The expression of surface molecules on unstimulated γδ T cells was determined by flow cytometry. On day 10 of culture, the degranulation assay and the cytotoxicity assay were performed. (**A**) Correlation between the percentage of CD16 or CD69 expressing unstimulated γδ T cells and the ΔMFI of CD107a on γδ T cells or the percentage of CD107a expressing γδ T cells after incubation with Daudi for 3h on day 10 of stimulation. (**B**) Correlation between the ΔMFI of CD25 on unstimulated γδ T cells and the percentage of CD107a expressing γδ T cells after incubation with Daudi for 3h on day 10 of stimulation. (**C**) Correlation between the percentage of CXCR3 expressing unstimulated γδ T cells and the lytic units (LU) per 10^6^ effector cells in the cytotoxicity assay with Daudi and rituximab on day 10 of stimulation. (**D**) The 8 independent experiments of (**C**) are divided into two equal groups based on the percentage of CXCR3^+^ unstimulated γδ T cells (> or <85%). The cytotoxicity of the stimulated γδ T cells in these two groups is compared based on the LU per 10^6^ effector cells obtained from the cytotoxicity assay with Daudi and rituximab performed on day 10 of culture. The data are presented as a correlation chart of 14 (**A**,**B**) or 8 (**C**) independent experiments or as mean ± SD of 8 (**D**) independent experiments. Correlation coefficient R is calculated according to Spearman. * *p* < 0.05, ** *p* < 0.01, and *** *p* < 0.001 correlating or comparing the indicated variables.

The question arises if we can already deduce from characteristics of the donors’ unstimulated γδ T cells whether their stimulated γδ T cells exhibit better anti-tumor activity. With regard to the degranulation at day 10, there is a negative correlation with the expression of CD16 and a positive correlation with the expression of CD69 and CD25 on unstimulated γδ T cells of 14 healthy donors (Figure 4A,B). For CD69, the correlation could also be seen if the degranulation assay has been performed with U2932 (Figure S5B). As expected, considering this result and Figure 3A, there is a significant positive correlation between the proportion of CD16 and CD69 expressing unstimulated γδ T cells and the proportion of CD16 and CD69 expressing stimulated γδ T cells at day 10 of culture with Ko-Op (Figure S6). The same applies for the expression of CD62L on unstimulated and stimulated γδ T cells (Figure S6). Next, we asked whether the phenotype of unstimulated γδ T cells could predict the ADCC of γδ T cells. Therefore, we performed a cytotoxicity assay with stimulated MNC from eight healthy donors consisting of >90% γδ T cells, the Burkitt lymphoma cell line Daudi and the monoclonal antibody rituximab or its corresponding isotype control (Figure S7). We found a statistically significant correlation between the percentage of CXCR3 expressing unstimulated γδ T cells and the lytic units per 10^6^ effector cells in the cytotoxicity assay with rituximab on day 10 of culture with Ko-Op (Figure 4C). To confirm that the expression of CXCR3 on the unstimulated γδ cells predicts the subsequent cytotoxicity of stimulated γδ cells, we divided the experiments of Figure 4C into two equal groups based on the percentage of CXCR3^+^ unstimulated γδ T cells (more or less than 85%). We found significantly higher lytic units per 10^6^ effector cells in the group with higher CXCR3 expression on unstimulated γδ cells (Figure 4D). Thus, the expression of individual surface molecules on the unstimulated γδ T cells can be used as a decision-making aid in the selection of donors.

### 3.4. Influence of the Cultivation Method on the Phenotype of Stimulated γδ T Cells

**Figure 5:**
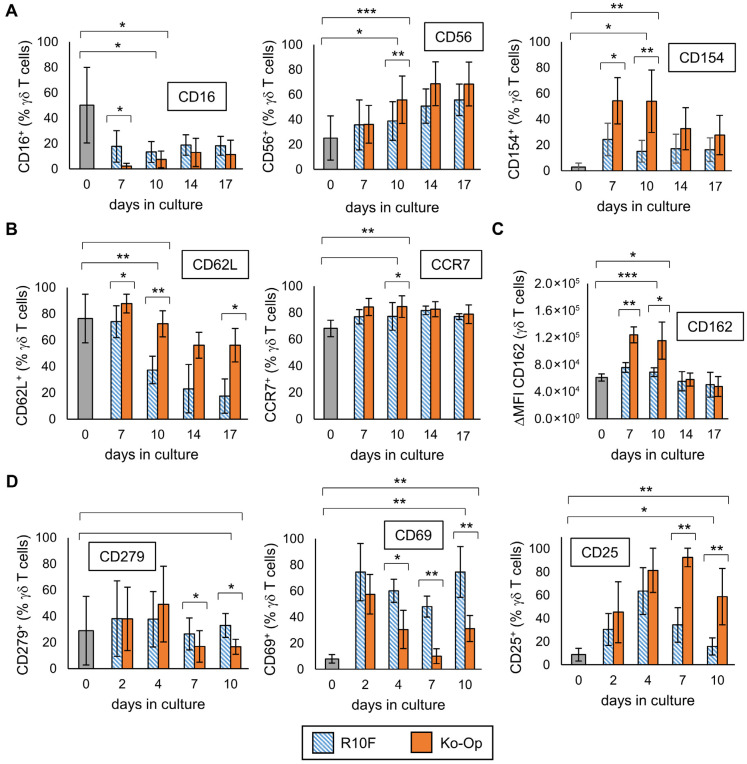
Influence of the cultivation method on the phenotype of γδ T cells. MNC of healthy donors were isolated and stimulated according to the protocols R10F (blue bars) and Ko-Op (orange bars) for up to 10 to 17 days. The surface molecules on γδ T cells at the different days of culture were determined by flow cytometry. (**A**) Percentage of three different surface molecules expressing γδ T cells at different days of culture. The molecules are markers for activity. (**B**) Percentage of two different surface lymph node homing molecules expressing γδ T cells at different days of culture. (**C**) Expression of the adhesion molecule CD162 (shown as ΔMFI) on γδ T cells at different days of culture. (**D**) Percentage of CD25, CD279, and CD69 expressing γδ T cells at different days of culture. The data are presented as mean ± SD of 4–5 (**A**–**C**) or 5–6 (**D**) independent experiments. * *p* < 0.05, ** *p* < 0.01, and *** *p* < 0.001 comparing the two different stimulation protocols and d0 and d10 for each protocol.

Next, we were interested in how the expression of surface molecules varies during culture and if changing the cultivation protocol leads to differences in the phenotype. Thus, MNC were isolated from five healthy donors and cultured with either Ko-Op or another stimulation protocol called R10F for 17 days. In the Ko-Op protocol a serum-free medium is used, whereas a medium containing fetal calf serum is used in the R10F protocol. Furthermore, the concentrations of zoledronate and IL-2 are higher in Ko-Op than in R10F, and the culture takes place in bottles with shaking from d4 onwards, whereas in R10F protocol the cells are cultured in 96-well plates. We have already shown that using the Ko-Op protocol leads to higher degranulation ability and ADCC of γδ T cells than R10F [12]. Figure 5A shows the results for three surface molecules with activity-related functions. On certain days, culture with Ko-Op results in a significantly higher proportion of CD56 (day 7) and CD154 (days 7 and 10) expressing γδ T cells and a significantly lower proportion of CD16 (day 7) expressing γδ T cells, with an overall decrease in the proportion of CD16 expressing γδ T cells compared to day 0. In addition, the modification of the cultivation protocol significantly enhanced the expression of surface molecules with the function of lymph node homing (Figure 5B). When cultured with Ko-Op, the proportion of CD62L expressing γδ T cells is higher on most days of the experiment and the proportion of CCR7 expressing γδ T cells at day 10. The expression of the adhesion molecule CD162 on γδ T cells is also increased on day 7 and 10 when cultured with Ko-Op (Figure 5C). Since CD25, as part of the IL-2 receptor, CD69, as marker of early activation, and CD279 (also known as PD-1), as regulator of the immune response, play a role from the beginning of stimulation, we cultured MNC from six healthy donors until day 10 and examined their expression every second to third day of culture. When cultured with Ko-Op, we observed a significantly increased proportion of CD25 expressing γδ T cells and decreased proportion of CD279 expressing γδ T cells from day 7 compared to stimulation with R10F. Furthermore, the proportion of CD69 expressing γδ T cells was decreased significantly from day 4 (Figure 5D). To complete the overview given in Figure 2A-C, we analyzed, for all the molecules tested in Figure 5, if the difference between unstimulated and stimulated γδ T cells (on day 10) is significant for the respective protocol. For CD28, NKG2D, CD27, CD44, CD103, LFA-1, CXCR3, CD45RA, and CD57 there is no significant difference in expression between the two cultivation methods. The change in the expression of these surface molecules over time during culture with Ko-Op is shown in Figure S8A+B. Further markers were tested on day 10 of culture with regard to their differences between the cultivation methods. Here, we observed increased expression of LAG-3 and CXCR4 after Ko-Op stimulation compared to R10F, while CD31 and CD161 were reduced in comparison (Figure S9). No differences were observed in the percentage of TIGIT^+^, TIM-3^+^, CCR2^+^, and DNAM-1^+^ γδ T cells. In summary, changing the stimulation protocol leads to significant changes in the phenotype of stimulated γδ T cells.

## 4. Discussion

Here we present an intensive analysis of the phenotype of γδ T cells from the peripheral blood of healthy donors. We investigated the extent to which stimulation with zoledronate and IL-2 influences this phenotype over the time and whether variations in the stimulation protocol may lead to further phenotypical modifications. Through correlation analyses with results from various experiments investigating the anti-tumor effects of stimulated γδ T cells against the three lymphoma cell lines, we were able to establish a link between surface molecule expression and function, thereby determining further criteria for selecting an ideal donor. In the following sections, we will discuss some individual surface molecules in detail.

We showed that the proportion of CD16 expressing γδ T cells significantly decreases under stimulation with both protocols. This is in contrast to the results of Kim et al., who saw no difference in CD16^+^ γδ T cells after stimulation with zoledronate, IL-2, and IL-15 [32]. Lafont et al. observed low expression of CD16 upon eight days of stimulation by nonpeptidic antigens and IL-2 with a consecutive increase reaching a plateau after approximately three weeks [39]. However, the stimulation protocols differ from ours, so a direct comparison is not possible. As CD16 is known to mediate ADCC, the question arises if the decrease in CD16 upon stimulation with Ko-Op is unfavorable for future cellular therapy. We already demonstrated that reduced CD16 expression does not correlate with reduced ADCC by γδ T cells and only a low expression of CD16 is necessary for ADCC [40]. Additionally, the monoclonal antibody obinutuzumab leads to an enhanced cytotoxicity against different B cell lymphoma cell lines by γδ T cells that are stimulated with the Ko-Op protocol indicating that the ADCC is not impaired [41]. Ryan et al. demonstrated that CD16^−^ γδ T cells still have the capacity to target cancer cells via degranulation of granzymes [42]. In line with this, we observed a negative correlation between the proportion of CD16 expressing γδ T cells and of the CD107a expressing γδ T cells in the degranulation assay with two lymphoma cell lines. We therefore conclude that the decrease in CD16 expression on γδ T cells mediated by stimulation with zoledronate and IL-2 is not a disadvantage but can be even associated with increased anti-lymphoma activity of stimulated γδ T cells.

CD25 (IL-2Rα) is a part of the IL-2 receptor and its expression on γδ T cells increases significantly upon stimulation with zoledronate and IL-2 as it has already been shown for activated γδ T cells [11,43]. While the increase on day 2 and 4 does not differ significantly between the two stimulation protocols, the percentage of CD25 expressing γδ T cells is significantly higher on day 7 and 10 when stimulated with Ko-Op. We assume that the addition of IL-2 can still have a stimulatory effect on day 7 and 10 leading to a higher proliferation rate on these days, while the sensitivity to IL-2 of γδ T cells stimulated with R10F decreases. For this reason, and because of the positive correlation between the proportion of CD25 expressing T cells and of the CD107a expressing T cells in the degranulation assay with lymphoma cell lines, the expression of CD25 on stimulated γδ T cells is an important parameter for further evaluation of a stimulation procedure.

We can show that with both cultivation methods, the proportion of CD69 expressing γδ T cells increases clearly up to day 2 of culture. Interestingly, while by stimulation with R10F the percentage of CD69^+^ γδ T cells remains continuously high, by stimulation with Ko-Op, the proportion of CD69 expressing γδ T cells already decreased significantly on day 4. The expression of CD69 is shown to negatively regulate the number of tumor-infiltrating T cells and is associated with CD8^+^ T cell exhaustion [44]. On day 10 of culture with Ko-Op, we observed a negative correlation between the proportion of CD69 expressing γδ T cells and the proliferation rate. Additionally, we know from our previous study that γδ T cells, which have been stimulated by R10F, proliferate less than γδ T cells stimulated with Ko-Op [12]. In consideration with the higher CD69 expression after R10F stimulation, these findings would support a link between CD69 expression and exhaustion. On the other hand, we observed a positive correlation between the proportion of CD69 expressing γδ T cells and the proportion of CD107a expressing γδ T cells after incubation with two lymphoma cell lines suggesting that CD69^+^ γδ T cells have higher capacity to degranulate in response to target cells. This contradicts the theory of exhaustion. Additionally, neither the expression of CD57 as a marker for senescence nor the expression of PD-1 as a marker for exhaustion seem to correlate with CD69 expression on freshly isolated γδ T cells of healthy donors and the lower proliferation rate of R10F cells does not necessarily indicate exhaustion. Another point is that we saw a higher expression of CD69 after stimulation with R10F here, but, in our previous paper, a lower expression of the degranulation marker CD107a compared to stimulation with Ko-Op [12]. Ultimately, the meaning of CD69 expression on stimulated γδ T cells can only be speculated on the basis of the available data and has to be investigated in further studies.

As part of this study, we also investigated activating NK cell receptors. While NKG2D and DNAM-1 were expressed by unstimulated peripheral γδ T cells and were upregulated under Ko-Op stimulation, we saw at most a slight expression of NKp30, NKp44, and NKp46 in either unstimulated or stimulated cells. This is consistent with the findings of Correia et al., who observed an increase in the expression of these NK cell receptors under stimulation with certain cytokines, but only in Vδ1 T cells and not in Vδ2 T cells [45].

It can be assumed that the stimulated γδ T cells, which are supposed to be efficient against solid tumors in vivo, must express adhesion molecules on their surface that allow the cells to migrate into the tumor tissue. We can demonstrate that the expression of most investigated adhesion molecules increase by stimulating γδ T cells with Ko-Op with a significantly higher expression of CD162 and CXCR4 compared to R10F stimulation. CXCR4, though, is expressed on substantially different levels depending on the stimulation protocol. While R10F led to a significantly decreased CXCR4 expression, Ko-Op resulted in an increased expression. That finding is even more remarkable, since Vδ2 T cells are regarded as CXCR4^dull^ in comparison to CXCR4^bright^ Vδ1 T cells, in general [46]. Consequently, CXCR3^bright^/CXCR4^dull^ Vδ2 T cells were shown to display faster transendothelial migration to the CXCR3-binding IP-10/CXCL10 than to the CXCR4-specific chemokine SDF-1. In turn, SDF-1 was more effective for CXCR3^dull^ and CXCR4^bright^ Vδ1 T cells [18]. Poggi et al. have assigned the expression of CD31 to Vδ1, and of CD161 to Vδ2 T cells as critical for their respective transendothelial migration [47]. In line with these insights, the expression of CD31 decreased on our cultured Vδ2 T cells in general. It is of note that the adhesive and signaling functions of CD31 are to be integrated in the immune and the vascular system as well. Therefore, the overall biological functions of CD31 seems to be complex, but—with regard to its functions in the immune system—recent studies associate CD31 expression on T cells as correlated to immunosenescence and reduced amounts of differentiated effector T cells [48,49,50]. Consequently, the Ko-Op-induced reduction in CD31 expression may be regarded as indication for a higher activity of γδ T cells against malignant cells and therefore beneficial to a further treatment option. CD161, on the other hand, was expressed on the same level on unstimulated and R10F-cultured γδ T cells, though its expression decreased on Ko-Op-cultured cells. While it was identified as critical for transendothelial migration of Vδ2 T cells (see above), CD161 was also recently used as marker to define human Vδ1^−^ γδ T cell subsets due to its correlation to central and effector memory T cells [51]. Gao et al. pointed out, that γδ T cells, iNKT cells, and MAIT cells express CD161 at a higher level compared to conventional T_h_ and T_c_ cells [52]. Provine et al. suggested CD161 expression as an indicator of a transcriptional program for the capacity of TCR-independent activation by IL-12 and IL-18—which results in the robust production of interferon γ [53]. Very recently, Tong et al. stated that due to the above mentioned innate-like response pattern, CD161 is a marker of human memory T cells with innate immune-like functional potential and the ability to generate IL-17A [54]. Taken together, the reduction in CD31 and CD161 on γδ T cells is consistent with our findings that γδ T cells cultured in Ko-Op are more likely to reveal an effector T cell phenotype. Whether the increase in other adhesion and migration molecules leads to increased migration into the tumor tissue must be investigated in future studies.

CD62L (L-Selectin) and CCR7 (CD197) are both expressed by naive γδ T cells and TCM, but downregulated on TEM [26]. Although in our study the proportion of TEM increased significantly upon stimulation, we saw a significant increase in CCR7^+^ γδ T cells and no decrease in the proportion of CD62L^+^ γδ T cells after ten days of stimulation with Ko-Op. Especially with regard to the expression of CD62L but also CCR7 we saw a clear difference between the two stimulation methods, with Ko-Op stimulation leading to a significantly higher expression of CD62L and, to a lesser extent, of CCR7 compared to R10F. Furthermore, we observed a positive correlation between the proportion of CD62L^+^ γδ T cells on day 10 of stimulation and the proliferation rate on day 10 suggesting that the CD62L^+^ γδ T cells retain their memory function. As a central memory subset expressing CCR7 and CD62L has been shown to be more effective at controlling tumor in vivo than an effector memory subset, we assume this is a clear benefit of using Ko-Op for γδ T cell expansion [28,29,30].

Although we saw changes in CCR7 and CD62L expression, we did not see a change in the proportion of TCM upon stimulation with Ko-Op. It is possible that the categorization based on CD45RA and CD27 is not precise enough to show relevant differences. The correlations between the expression of CD57 and CD28 and the percentage of TEMRA and TCM on unstimulated γδ T cells presented here show at least that the classification of γδ T cells on the basis of CD45RA and CD27 is at this point valid with regard to function. Indeed, CD57 is a marker of differentiated cells that no longer proliferate. Expectedly, the proportion of CD57 expressing γδ T cells correlates positively with the proportion of TEMRA and negatively with the proportion of TCM [55]. CD28, in contrast, acts as a co-stimulator and is known to be involved in a long-sustaining immune response of T cells [56]. Furthermore, the CD28/B7 costimulatory signals are involved in the proliferation of human γδ T cells via IL-2 [57]. CD28^−^ T cells are characterized as highly differentiated T cells [58]. Accordingly, we could show that the percentage of CD28 expressing γδ T cells negatively correlates with the proportion of CD57 expressing γδ T cells and of TEMRA and positively with the proportion of TCM.

In this study, we also investigated the expression of various immune checkpoints. Even though other authors have observed an increase in PD-1 expression when γδ T cells were stimulated by phosphoantigens, we do not observe any changes [59]. This is consistent with reports showing that PD-1 expression on healthy adult peripheral Vγ9Vδ2 T cells is generally low, rising only briefly after activation and then rapidly declining [60]. Therefore, the changes in the expression are considered rapid and transient, present in the initial steps of stimulation, and do not appear to be relevant for the final cell product. On the other hand, the stimulation of γδ T cells leads to an upregulation of TIGIT, LAG-3, and TIM-3. This may occur as a regulatory negative-feedback mechanism triggered by extensive γδ T cell activation. Since these immune checkpoints are associated with immune cell exhaustion and reduced anti-tumor response, the question arises as to whether this increase is detrimental to future in vivo cancer therapy with these cells [61]. Interestingly, we found a positive correlation between TIM-3 expression and degranulation in presence of different lymphoma cell lines what is not consistent to the finding that TIM-3 leads to reduced anti-tumor responses of Vγ9Vδ2 T cells against colon cancer cells [62]. You et al. observed enhanced anti-tumor activity of TIGIT^+^ γδ T cells compared to their TIGIT^−^ counterparts, which is also in contrast to the paradigm of immune checkpoint function and patient derived data [63,64]. Another interesting finding is that the co-localization of the T cell receptor and LAG-3 was determined to play a significant part in the role of immune inhibition, indicating that its function is not defined merely by upregulation [65]. These findings indicate that the regulatory function of immune checkpoints on γδ T cell is complex, context dependent, varies with differentiation state and disease environment, and warrants further investigation also with regard to future in vivo application.

In addition to the surface molecules, we investigated the morphology of stimulated compared to unstimulated γδ T cells. The increase in size and the irregular conformation resemble the phenotype of activated and migratory T cells [66,67,68]. Activated γδ T cells must be able to move, to recognize the antigen-presenting cells, and form an immunological synapse with them. The cytoskeletal actin plays a crucial role for that [69,70]. We also observed the aggregation of stimulated γδ T cells, which is in concordance with the homotypic aggregation of stimulated αβ T cells via LFA-1/ICAM-1 as part of the immunological synapse [71,72]. In line with this, we found an increase in activated LFA-1 after stimulation of γδ T cells. Therefore, the morphological changes presented here might be relevant for the anti-tumor activity of γδ T cells.

Adoptive transfer of haploidentical γδ T cells is feasible without relevant side effects. Therefore, the question of a suitable donor arises as we see inter-person variability in terms of yield and anti-tumor activity when stimulating their MNC ex vivo. We have already demonstrated that the youngest adult donor with the highest percentage of γδ T cells within the unstimulated MNC is preferable [12]. Now, we evaluated the phenotype of unstimulated γδ T cells in relation to the proliferation rate, cytotoxicity, and CD107a expression in the degranulation assay after 10 days of stimulation. The negative correlation between CD16 expression and CD107a expression may be due to the higher expression of CD16 on TEMRA, which have been shown not to respond to stimulation with phosphoantigens in terms of IFNy and TNFα production [73]. Interestingly, Lee et al. found that high expression of CD16 is beneficial for Vδ2 T cell engineering for the treatment of ovarian cancer [74]. However, in comparison to our study, other assays and cell lines were used here to determine anti-tumor activity. Furthermore, CD69 expression on unstimulated γδ T cells, on the other hand, appears to be beneficial for degranulation capacity on day 10. It is possible that in donors with a high proportion of CD69^+^ γδ T cells, these cells are already pre-activated, making them more amenable to stimulation. However, we also saw that the proportion of CD69^+^ γδ T cells at day 10, which correlates with the proportion of unstimulated CD69^+^ γδ T cells, is associated with a decreased proliferation rate. In addition, the expression of CD25 on unstimulated γδ T cells correlates with degranulation at day 10. As CD25 is part of the IL-2 receptor, high amounts of CD25 on γδ T cells probably make them more sensitive to stimulation with IL-2. Considering the ADCC, we observed a positive correlation with CXCR3 expression on unstimulated γδ T cells. The negative correlation between age and CD62L expression on unstimulated γδ T cells substantiates our previous finding that γδ T cells from older people are not as easily stimulated as those from younger individuals [12]. Indeed, this finding correlates positively with CD62L expression on stimulated γδ T cells, which seems to be associated with a better proliferation rate. In summary, our data indicate that the following characteristics of donors and their γδ T cells may be suitable for selecting the best available donor: younger age, a higher percentage of γδ T cells, fewer CD16^+^ γδ T cells, and more CD62L^+^ and CXCR3^+^ γδ T cells. Additionally, a higher level of CD25 on γδ T cells is favorable. However, it should be noted that the correlations between CD16, CD25, or CXCR3 expression and anti-tumor function were observed by investigating γδ T cells’ activity against the Daudi cell line. In degranulation assays with other lymphoma cell lines, no correlation between CD16 and CD25 expression and CD107a expression has been found. This should be taken into account when evaluating the criteria in the future.

With this study, we present an intensive analysis of the differential expression of surface molecules of γδ T cells from the peripheral blood of healthy donors. We investigated to what extent stimulation with zoledronate and IL-2 influences their phenotype over the time and whether changes in the stimulation protocol may lead to further phenotypical modifications. Through correlation analyses with results from various experiments in which the anti-tumor effects of stimulated γδ T cells were investigated, we were able to draw a link between surface molecule expression and function and determined additional criteria for an ideal donor. Further studies including migration experiments are necessary to prove if the stimulation with Ko-Op and the associated phenotypic changes also lead to enhanced anti-tumor activity and migration capacity in vivo.

## Data Availability

The datasets generated for this study are available on request to the corresponding author.

## Supplementary Materials

The following supporting information can be downloaded at: https://www.mdpi.com/article/10.3390/cells14231917/s1, Figure S1: Flow cytometry of unstimulated and stimulated MNC; Figure S2: Flow cytometry of unstimulated and stimulated γδ T cells; Figure S3: Flow cytometry of unstimulated and stimulated γδ T cells; Table S1: Correlation between the expression of the individual surface molecule; Figure S4: Degranulation of stimulated γδ T cells; Figure S5: Correlations between CD107a expression and surface molecule expression on unstimulated and stimulated γδ T cells; Figure S6: Correlation between phenotype of unstimulated and stimulated γδ T cells; Figure S7: Antibody-dependent cellular cytotoxicity of stimulated γδ T cells; Figure S8: Changes in the expression of individual surface molecules on γδ T cells over time; Figure S9: Differences in the expression of individual surface molecules on γδ T cells after stimulation with R10F or Ko-Op.

## Abbreviations

The following abbreviations are used in this manuscript:

APC	allophycocyanin
ECD	r-Phycoerythrin-Texas Red
DNAM-1	DNAX Accessory Molecule-1
FITC	fluorescein isothiocyanate
GMP	Good Manufacturing Practice
IFNy	interferon y
IL-2	interleukin 2
LAG-3	lymphocyte-activation gene 3
LFA-1	lymphocyte function-associated antigen 1
LU	lytic units
MHC	major histocompatibility complex
MFI	median fluorescence intensity
MNC	mononuclear cells
NK cells	natural killer cells
NKG2D	natural killer group 2 member D
PB	pacific blue
PC5	phycoerythrin-cyanine 5
PC7	phycoerythrin-cyanine 7
PE	phycoerythrin
TCM	central memory γδ T cells
TEM	effector memory γδ T cells
TEMRA	terminally differentiated γδ T cells
TIGIT	T cell immunoreceptor with Ig and ITIM domain
TIM-3	T cell immunoglobulin and mucin-domain containing 3
