# Supplementary material for: Characterization of Stimulated γδ T Cells: Phenotypic Analysis and Implications for Allogeneic Cellular Immunotherapy
Source: Cells. 2025 Dec 2;14(23):1917. doi: 10.3390/cells14231917 (PMC12691411; doi:10.3390/cells14231917)
Supplement: Supplementary file 1 [file cells-14-01917-s001.zip › cells-3912445-supplementary.pdf]

Figure S1

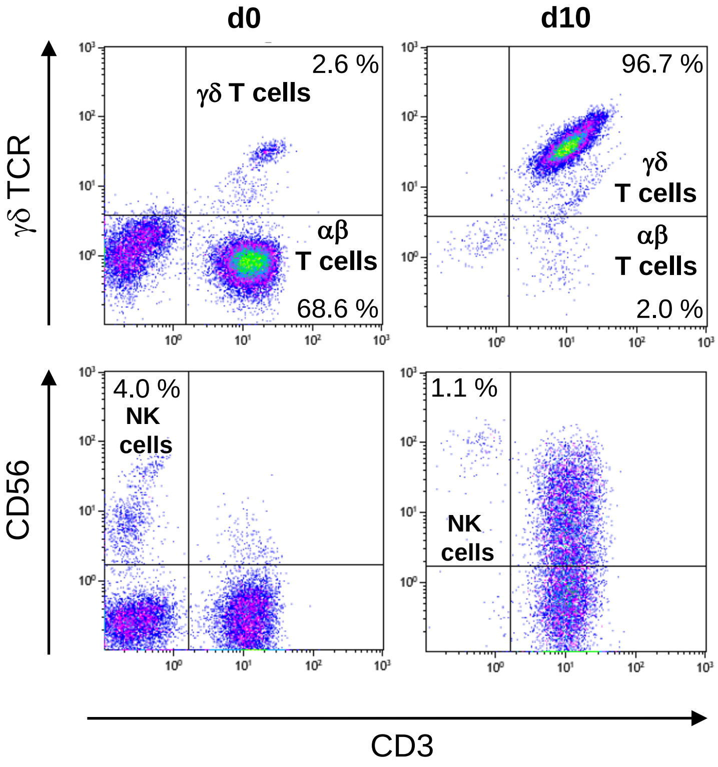

Flow cytometry of unstimulated and stimulated MNC
MNC of a healthy donor were isolated and stimulated according to the Ko-Op protocol. Exemplary gating strategy is shown for determination of γδ T cells, αβ T cells and NK cells by flow cytometry at day 0 and day 10.

Figure S2

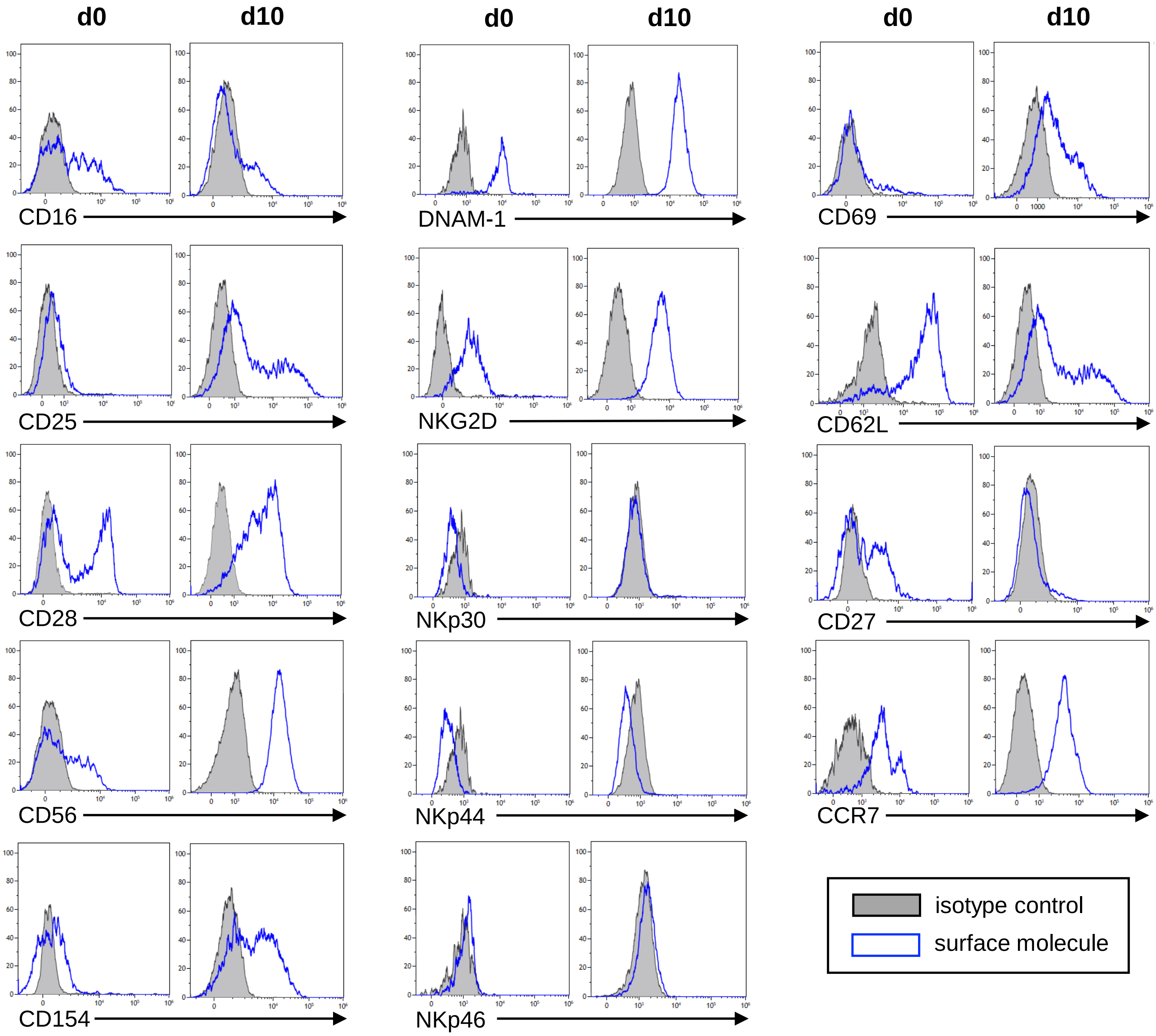

Flow cytometry of unstimulated and stimulated γδ T cells
MNC of healthy donors were isolated and stimulated according to the Ko-Op protocol. Exemplary histograms for γδ T cells' expression of surface molecules associated with activity and lymph node homing at day 0 and day 10 of culture examined by flow cytometry are shown.

Figure S3

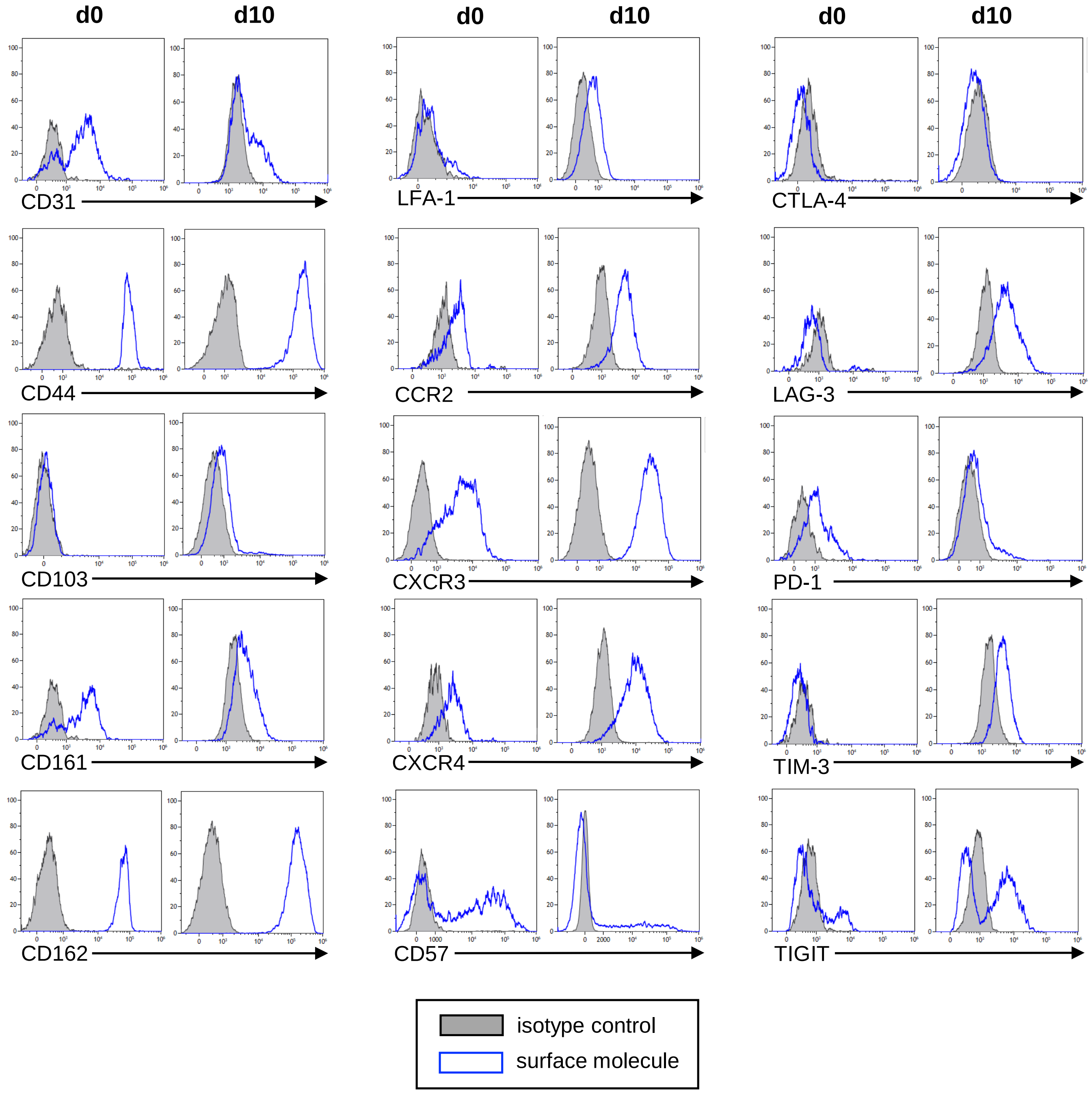

Flow cytometry of unstimulated and stimulated γδ T cells
MNC of healthy donors were isolated and stimulated according to the Ko-Op protocol. Exemplary histograms for γδ T cells‘ expression of surface molecules associated with adhesion, migration and regulation at day 0 and day 10 of culture examined by flow cytometry are shown.

Table S1

A

	Naive (% γδ T cells)	TCM (% γδ T cells)	TEM (% γδ T cells)	TEMRA (% γδ T cells)
CD16+ (% γδ T cells)				0.54 (*)
CD28+ (% γδ T cells)		0.67 (**)		-0.87 (***)
CD57+ (% γδ T cells)		-0.63 (**)		0.82 (***)

B

ΔMFI		activity						adhesion/migration					lymph node homing		other		
		CD16	CD25	CD28	CD56	NKG2D	CD154	CXCR3	LFA-1	CD44	CD103	CD162	CD69	CCR7	CD57	CD279	CD45RA
activity	CD16			-0.62 (**)		0.73 (***)			0.55 (*)	0.51 (*)					0.46 (*)		
	CD25			0.71 (***)				0.68 (**)	-0.49 (*)						-0.52 (**)		-0.56 (**)
	CD28	-0.62 (**)	0.71 (***)			-0.52 (*)	0.56 (**)	0.62 (**)	-0.50 (*)						-0.82 (**)		-0.72 (**)
	CD56								0.58 (**)								0.54 (*)
	NKG2D	0.73 (***)		-0.52 (*)					0.65 (**)	0.71 (**)		0.50 (*)			0.46 (*)		
	CD154			0.56 (**)				0.55 (*)			0.72 (***)						
adhesion/ migration	CXCR3		0.68 (**)	0.62 (**)			0.55 (*)								-0.51 (*)		-0.56 (*)
	LFA-1	0.55 (*)	-0.49 (*)	-0.50 (*)	0.58 (**)	0.65 (**)						0.60 (**)			0.49 (*)		
	CD44	0.51 (*)				0.71 (**)							0.67 (**)				
	CD103						0.72 (***)										
	CD162					0.50 (*)			0.60 (**)						0.46 (*)	0.56 (*)	
lymph node homing	CD69									0.67 (**)							
	CCR7															0.71 (**)	
other	CD57	0.46 (*)	-0.52 (**)	-0.82 (**)		0.46 (*)		-0.51 (*)	0.49 (*)			0.46 (*)					0.73 (**)
	CD279											0.56 (*)		0.71 (**)			
	CD45RA		-0.56 (**)	-0.72 (**)	0.54 (*)			-0.56 (*)							0.73 (**)		

Correlation between the expression of the individual surface molecules

MNC of healthy donors were isolated. The percentage of CD16, CD28 and CD57 expressing γδ T cells and of the naive, TCM, TEM and TEMRA (A) as well as the ΔMFI of the surface molecules on γδ T cells (B) were determined by flow cytometry. Correlation coefficient R is calculated according to Spearman and written in the tables. The positive correlations are coloured green, the negative ones red. If there is no statistically significant correlation, the field is marked grey.

(A) The table shows the significant correlations between the proportion of the respective surface molecule expressing γδ cells and the proportion subgroup of the γδ T cells.

(B) The red and green boxes show the significant correlations between the ΔMFI of the individual surface molecule on γδ T cells.

The data were obtained from 19 (A) or 18-24 (B) independent experiments. *p<0.05, **p<0.01, ***p<0.001 correlating the indicated variables.

Figure S4

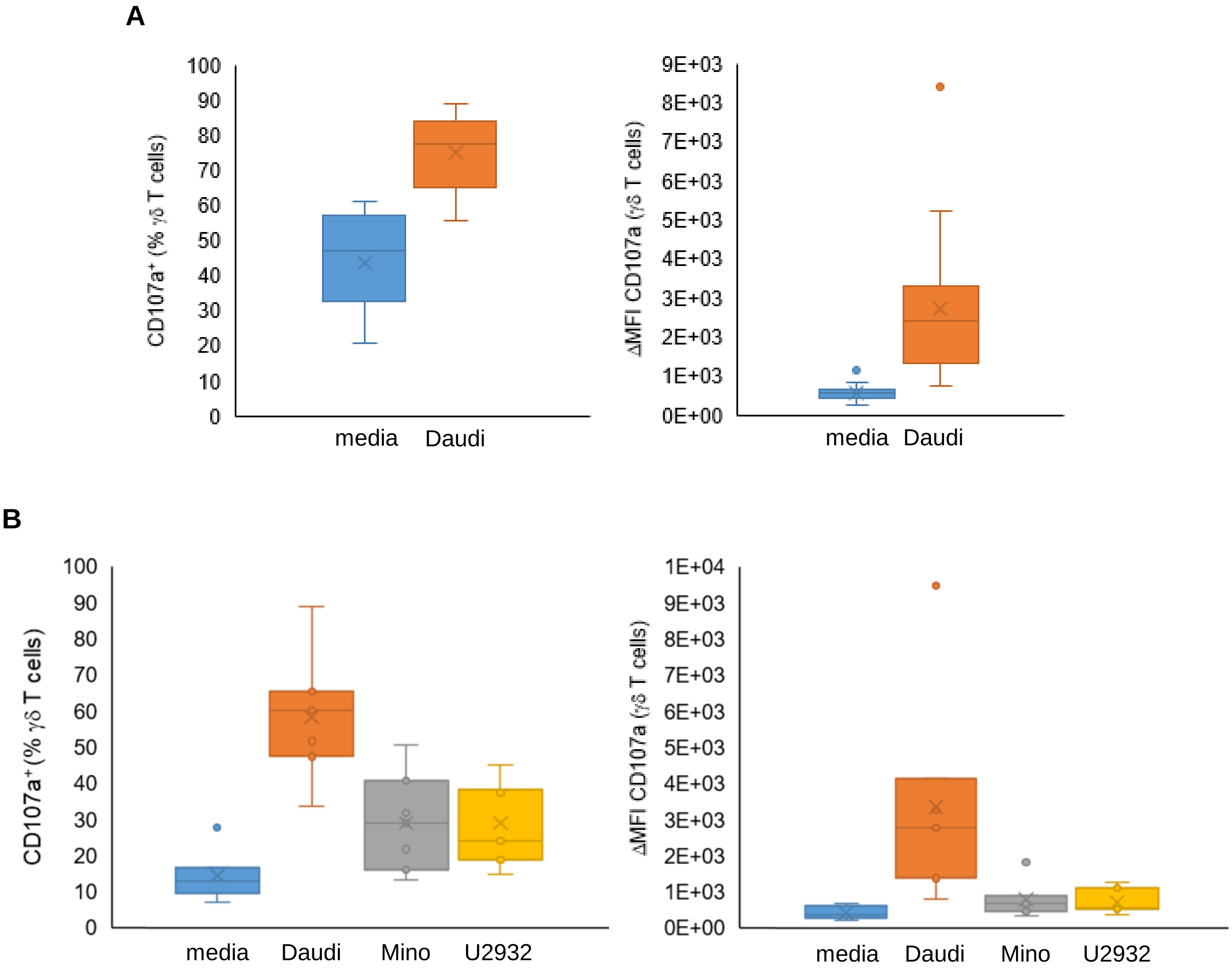

Degranulation of stimulated γδ T cells

MNC of healthy donors were isolated and stimulated according to the Ko-Op protocol. At day 10, MNC were incubated with media control or Daudi (A) or with media control, Daudi, Mino or U2932 (B) in order to perform the degranulation assay. After 3h, CD107a^{+} γδ T cells and ΔMFI of CD107a on γδ T cells were detected by flow cytometry.

The data are presented as boxplots of 14 (A) or 7 (B) independent experiments.

Figure S5

A

	CD107 expression (media)	CD107 expression (Daudi)	CD107 expression (Mino)	CD107 expression (U2932)
CD16 expression		-0.84 (***)		-0.82 (*)
CD69 expression		0.74 (**)	0.79 (*)	
TIM-3 expression	0.93 (**)	0.93 (**)	0.93 (**)	0.79 (*)
CD25 expression	0.69 (*)	0.79 (*)	0.86 (*)	0.86 (*)

B

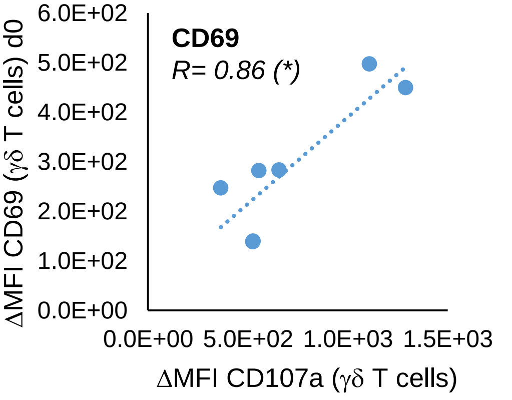

Correlations between CD107a expression and surface molecule expression on unstimulated and stimulated γδ T cells

MNC of healthy donors were isolated and stimulated according to the Ko-Op protocol. The expression of surface molecules on γδ T cells was determined on day 0 and d10 by flow cytometry. On day 10 of culture, a degranulation assay with media control, Daudi, Mino or U2932 was performed.

(A) The table shows the significant correlations between the expression of the respective surface molecule (percentage of γδ T cells or ΔMFI on γδ T cells) on day 10 and the expression of CD107 (percentage of γδ T cells or ΔMFI on γδ T cells) after incubation with media, Daudi, Mino or U2932. Correlation coefficient R is calculated according to Spearman and written in the tables. The positive correlations are coloured green, the negative ones red. If there is no statistically significant correlation, the field is marked grey.

(B) Correlation between the ΔMFI of CD69 on unstimulated γδ T cells and the ΔMFI of CD107a on γδ T cells after incubation with U2932 for 3h on day 10 of stimulation. The data are presented as correlation chart of 7 independent experiments. Correlation coefficient R is calculated according to Spearman. *$p<0.05$ correlating the indicated variables.

Figure S6

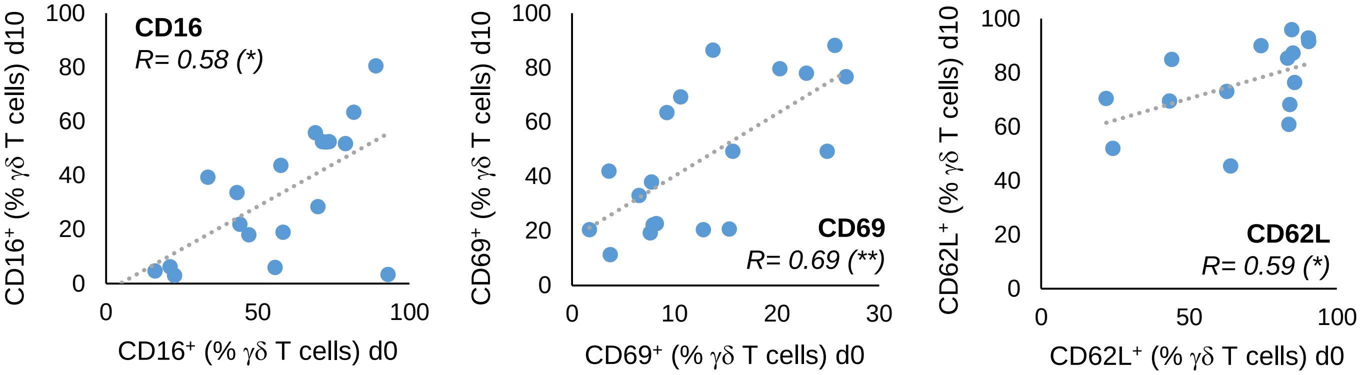

Correlation between phenotype of unstimulated and stimulated γδ T cells
MNC of healthy donors were isolated and stimulated according to the Ko-Op protocol. Surface molecule expression was determined by flow cytometry on unstimulated γδ T cells and on day 10 of culture. Correlation charts between the percentage of CD16, CD69 and CD62L expressing unstimulated (d0) and stimulated (d10) γδ T cells are shown. Correlation coefficient R is calculated according to Spearman.
The data were obtained from 15-19 independent experiments. *p<0.05, **p<0.01 correlating the indicated variables.

Figure S7

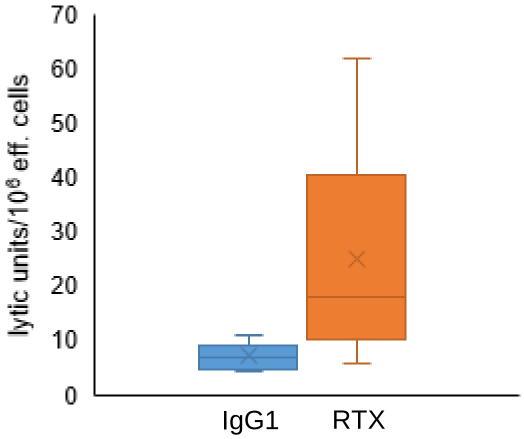

Antibody-dependent cellular cytotoxicity of stimulated γδ T cells
MNC of healthy donors were isolated and stimulated according to the Ko-Op protocol. At day 10 of culture, the stimulated MNC were co-cultured with Daudi and the monoclonal antibody rituximab (RTX) or its correspoding isotype control (IgG1) in order to perform the cytotoxicity assay. Lytic units per 10^6 effector cells were calculated based on the specific target cells lysis obtained with different effector to target ratios from 0.7:1 to 20:1. The data are presented as boxplots of eight independent experiments.

Figure S8

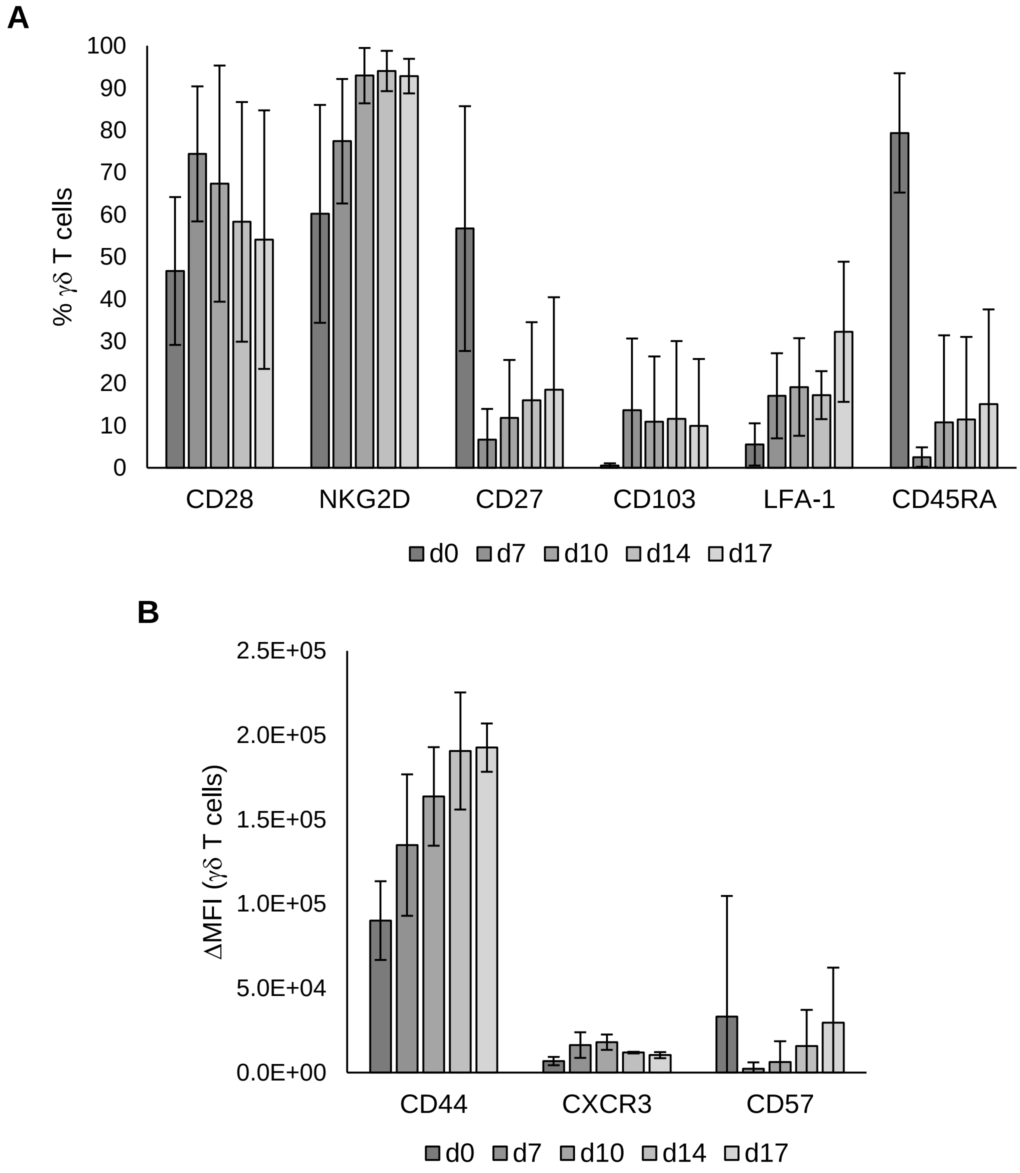

Changes in the expression of individual surface molecules on γδ T cells over time

MNC of healthy donors were isolated and stimulated according to the Ko-Op protocol. The expression of surface molecules on γδ T cells at different days of culture was determined by flow cytometry.

(A) Percentage of the different surface molecule expressing γδ T cells at different days of culture.

(B) Expression of the surface molecules CD44, CXCR3 and CD57 (shown as ΔMFI) on γδ T cells at different days of culture.

The data are presented as mean ± SD of 4-5 independent experiments.

Figure S9

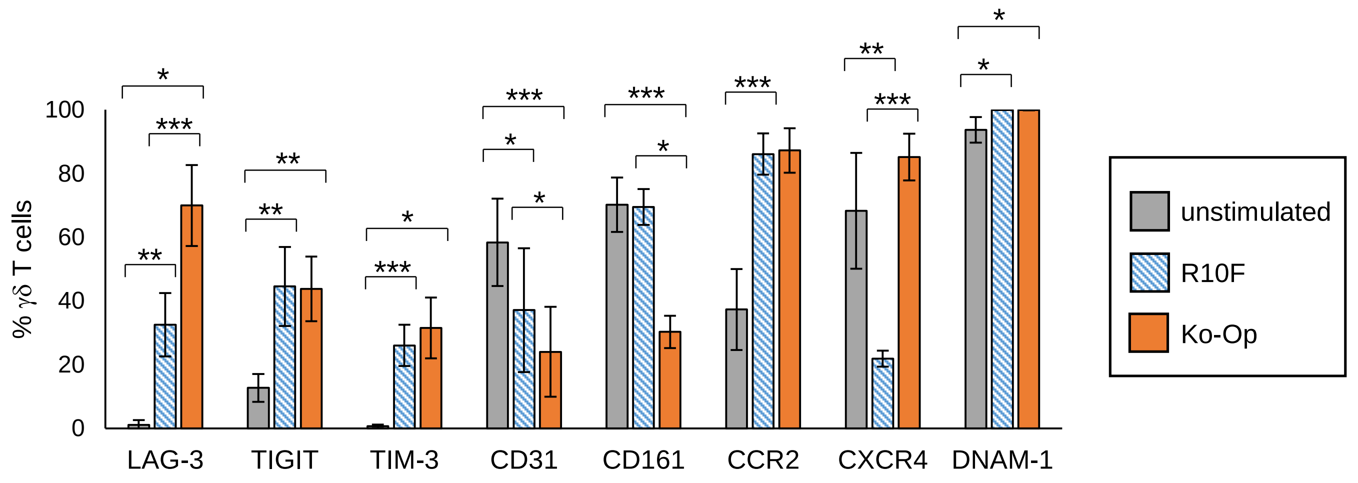

Differences in the expression of individual surface molecules on γδ T cells after stimulation with R10F or Ko-Op

MNC of healthy donors were isolated and stimulated according to the Ko-Op or R10F protocol. The expression of surface molecules on unstimulated γδ T cells at the day of isolation or at day 10 of culture with Ko-Op or R10F was determined by flow cytometry. The percentage of the respective surface molecule expressing γδ T cells is shown. The data are presented as mean ± SD of 5-6 independent experiments.
